# Experimental data on filtration–consolidation dewatering kinetics of different cassava flours

**DOI:** 10.1016/j.dib.2024.110600

**Published:** 2024-06-09

**Authors:** L. Van Der Werf, A. Chiadò Rana, A. Chapuis, C. Delpech, C. Wisniewski, F. Courtois

**Affiliations:** a Cirad, UMR QualiSud, F-34398 Montpellier, France; b Qualisud, Univ Montpellier, Avignon Université, Cirad, Institut Agro, IRD, Université de La Réunion, Montpellier, France; c Cirad, UMR QualiSud, Saint-Louis, Sénégal

**Keywords:** Compression, Cassava, Filtration, Kinetic, Dewatering

## Abstract

Dewatering is a critical step in cassava flours processing. Compression dewatering kinetics are useful to understand and design a dewatering operation. The dataset presents dewatering kinetics measured in a filtration–consolidation cell at constant pressure between 4 and 21 bar, on several cassava mashes (three batches fragmented at two particle size distributions (PSDs)). The dataset comprises, for each dewatering kinetic measurement, filtrate mass, cake height, data to estimate the pressure applied on the product (i.e. air pressure, compression force) as a function of time; and the moisture content measurements of the fresh and dewatered cassava and of the filtrate. A commented python script is included to read the dewatering experimental files and plot the kinetics Furthermore, the dataset extends its utility by including particle size distributions (PSDs) obtained from six cassava batches, subjected to several protocol variants. These data are useful for understanding the phenomena involved in cassava dewatering. They also serve as a valuable resource for researchers, designers, and operators to design cassava dewatering.

Specifications Table

**Table:** 

Subject	Food Science : Food Technology
Specific subject area	Food processing, solid–liquid separation, dewatering of cassava flour
Type of data	Tables
Data collection	Dewatering kinetics were measured in a filtration–consolidation cell. The cassava height, the filtrate mass, the air pressure applied on the jack and the strength applied on cassava were measured and recorded over time. The moisture content of cassava before and after dewatering, and that of the filtrate, were measured in an oven as recommended by the AOAC [1]. The particle size distributions (PSDs) of the fresh (i.e. not dewatered) rasped cassava were measured by spray sieving, as suggested by [2,3]
Data source location	Cirad (Centre International de Recherche en Agronomie pour le Développement), Montpellier, France
Data accessibility	Repository name [12]: Experimental filtration/compression dewatering kinetics of different cassava flours Data identification number: UNF:6:X2c3FhEibotKvzP98N0mPw== [fileUNF] Direct URL to data: https://doi.org/10.18167/DVN1/EVMJY2
Related research article	*L. Van Der Werf, A. Chiadò Rana, A. Chapuis, C. Delpech, C. Wisniewski, F. Courtois. Experimental study and modelling of a filtration–consolidation step: Towards the development of a design tool for cassava dewatering, J. Food Eng. 342 (2023).*https://doi.org/10.1016/j.jfoodeng.2022.111338

## 1 Value of the Data

• The dataset is useful to understand the mechanisms involved in cassava dewatering and to improve the performance of this operation.
• The dataset may be used for model fitting and validation. For this purpose, kinetics data were structured to be readable by a python script, making it easier to analyze them or to fit them to a model. Adding supplementary kinetics (generated experimentally or found in the literature) may lead to a more generic model.
• With the dataset, researchers, designers or operation managers can estimate the performance of cassava compression dewatering depending on the pressure applied and the product load.
• PSD measurement method, i.e. spray sieving, is not normalized. With the PSD sub-dataset, researchers are able to evaluate the repeatability, the reproducibility of the measurement and the sensitivity of several modalities (e.g. spraying time) on the measurement.
• The analysis of the data and their comparison to other similar product could help researchers understanding the influence of the matrix properties on its behavior during dewatering.

## 2 Background

Dewatering is a critical step in cassava flour processing, as in other food processing. Experiments in a filtration–consolidation cell are useful to better understand and optimize this operation [4,5]. No such experiments on cassava flour are available in the literature. This dataset was then generated to develop a design tool for cassava dewatering process. The tool aiming to be valid in several contexts, experiments were conducted on various cassava flour qualities.

In a first published paper [6], the dataset was used (i) to evaluate the performance of the compression dewatering of cassava and (ii) to fit a semi-empirical filtration–consolidation model based on both Hermia and Shirato et al. [7] models. The data article adds value to this first research article by making the kinetics available for future model improvement. Moreover, it provides data about the preliminary study of PSD measurement protocol adaptation and rasping repeatability. Therefore, one can continue the study with an in-depth understanding of the kinetics. In future works, both the predictive power of the model and its genericity could be improved. This dataset could then be completed with dewatering kinetics measured under other conditions, on various cassava qualities (origin, processing level, PSD), or even on other food products.

## 3 Data Description

Data are structured in two sub-datasets: one with the dewatering kinetics data; one with the particle size distributions (PSDs). Dewatering kinetics were measured on 3 batches of cassava, numbered from 1 to 3. PSDs were measured on the batch 1, also used for dewatering kinetics, and 5 other cassava batches, numbered from 4 to 8. Details about the batches are presented in the section “Materials and Methods”.

### 3.1 Dewatering kinetics dataset

Dewatering kinetics were measured in a filtration–consolidation cell between 4 and 21 bar. 3 batches of cassava, grated at 2 particle size distributions (PSDs), were dewatered. 2 graters were selected to obtain PSDs close to the ones found in processing units [3,8] (details in section Material and Methods). Kinetics were used in a first research article to fit and to validate a model [6]. Table 1 summarizes the conditions of the dewatering experiments conducted and available in the repository. It is also available at the root of the repository folder.

**Table 1:** Summary of dewatering experiments (available in repository: summary_kinetics.xlsx).

P	Batch	d_50_	Defrost	m_0_	Dry matter content *%, w.b.*			Mass balance error %	Folder name in the repository
bar		µm		g	DM_0_	DM_∞_	DM_filtr_		
4	1	700	yes	200.9	31.3	57.8	3.6	0.2	CASSAVA_P1.4_200g_210715_01
4	1	700	yes	200.7	36.2	59.0	5.6	2.2	CASSAVA_P1.4_200g_210719_01
4	1	1000	yes	200.1	36.0	60.1	5.6	1.3	CASSAVA_P1.4_200g_210803_01
4	1	1000	yes	200.0	36.3	56.2	5.8	9.2	CASSAVA_P1.4_200g_210804_01
4	1	1000	yes	101.0	36.6	57.5	5.5	0.4	CASSAVA_P1.4_100g_210819_01
4	1	1000	yes	100.8	36.6	54.9	5.5	3.7	CASSAVA_P1.4_100g_210920_01
6	1	700	yes	201.7	36.4	58.8	4.3	1.5	CASSAVA_P2.4_200g_210709_01
6	1	700	yes	201.7	35.5	59.3	5.6	2.3	CASSAVA_P2.4_200g_210713_01
6	1	1000	yes	201.7	34.9	57.2	5.5	0.6	CASSAVA_P2.4_200g_210813_01
6	1	700	yes	101.6	34.9	60.5	5.7	3.1	CASSAVA_P2.4_100g_210823_01
6	1	1000	yes	101.0	35.3	57.4	5.9	2.3	CASSAVA_P2.4_100g_210927_01
9	2	700	yes	401.4	42.8	64.3	8.7	1.9	CASSAVA_P3.8_400g_210607_02
9	2	700	yes	401.3	42.3	62.0	9.7	4.2	CASSAVA_P3.8_400g_210608_01
9	2	700	no	402.6	41.7	61.3	9.3	5.4	CASSAVA_P3.8_400g_210609_01
9	1	700	yes	201.7	36.9	59.3	5.5	-1.8	CASSAVA_P3.8_200g_210812_01
13	1	700	yes	200.8	31.8	62.9	5.9	2.8	CASSAVA_P5.67_200g_210720_01
13	1	700	yes	200.3	35.6	63.1	5.8	1.1	CASSAVA_P5.67_200g_210721_01
13	1	700	yes	100.8	33.9	62.5	5.7	4.6	CASSAVA_P5.67_100g_210723_01
13	1	700	yes	100.8	31.2	62.6	5.7	4.6	CASSAVA_P5.67_100g_210730_01
13	1	1000	yes	200.4	34.6	61.1	5.6	1.9	CASSAVA_P5.67_200g_210805_02
13	1	1000	yes	200.9	34.6	60.8	5.7	5.9	CASSAVA_P5.67_200g_210806_01
13	1	700	yes	201.1	36.2	63.0	6.0	2.0	CASSAVA_P5.67_200g_210809_01
13	1	1000	yes	200.6	35.9	65.3	5.7	0.7	CASSAVA_P5.67_200g_210817_01
15	3	700	no	201.2	37.4	62.8	8.8	2.5	CASSAVA_P6.67_200g_210621_01
15	3	700	yes	201.2	36.5	65.7	4.2	1.7	CASSAVA_P6.67_200g_210622_01
15	3	700	yes	200.2	39.2	66.5	8.3	4.0	CASSAVA_P6.67_200g_210623_01
16	1	700	yes	200.0	35.7	64.5	5.7	1.6	CASSAVA_P7.09_200g_210804_02
16	1	1000	yes	201.6	35.6	65.1	5.7	4.3	CASSAVA_P7.09_200g_210811_01
16	1	1000	yes	201.6	35.6	65.9	5.9	3.9	CASSAVA_P7.09_200g_210816_01
19	1	700	yes	200.3	35.0	65.8	7.8	4.2	CASSAVA_P8.5_200g_210809_02
19	1	1000	yes	200.2	35.4	64.5	5.8	3.5	CASSAVA_P8.5_200g_210722_01
19	1	1000	yes	199.9	32.3	66.8	5.7	1.8	CASSAVA_P8.5_200g_210812_02
21	1	700	yes	199.8	35.8	67.5	4.5	4.4	CASSAVA_P9.45_200g_210707_01
21	1	700	yes	199.8	35.4	65.3	4.5	2.6	CASSAVA_P9.45_200g_210707_02
21	1	1000	yes	201.7	36.0	65.5	5.6	3.7	CASSAVA_P9.45_200g_210726_01
21	1	1000	yes	199.5	35.0	65.3	5.8	5.5	CASSAVA_P9.45_200g_210802_01
21	1	700	yes	200.4	37.6	66.1	5.7	1.3	CASSAVA_P9.45_200g_210805_01
21	1	1000	yes	200.4	33.6	64.1	5.8	2.3	CASSAVA_P9.45_200g_210810_02
21	1	1000	yes	200.7	34.4	67.0	5.8	1.6	CASSAVA_P9.45_200g_210816_02
21	1	700	yes	201.7	35.4	67.0	5.8	3.5	CASSAVA_P9.45_200g_210818_01

With P the pressure applied on the product; d_50_ the median diameter, characterizing the product PSD here; “Defrost” indicates if the sample was deep-frozen and defrost before dewatering (yes) or dewatered quickly after rasping (no); m_0_ the initial mass of mash; DM_0_, DM_∞_, DM_filt_ respectively the dry matter content of the mash before and after dewatering, and of the filtrate; and the mass balance error estimated as $\frac{m_{0}-m_{\infty }-m_{filtr}}{m_{0}}$.

The repository contains one folder per kinetic. The name of the folder is coded as follow: 1_2_3_4_5_6_7 with (1) the product, (2) the air pressure in bar^1^, (3) the product initial mass in g, (4) the date of the experiment in the form yymmdd, (5) the trial number on this date (e.g. CASSAVA_P1.4_200g_210719_01).

Each folder contains three .txt files presenting the raw experimental data. The file named foldername_CIN (e.g. CASSAVA_P1.4_200g_210719_01_CIN) presents the raw measurements recorded by the sensors of the cell during the dewatering (e.g. filtrate mass, air pressure, see section material and methods). The file named foldername_DRY_MATTER presents the raw data of the moisture content measurement of the filtrate and of the product before and after dewatering (i.e. mass of the cup and of the sample before and after drying). The file named foldername_INFO presents general information about the experiment (e.g. operator name). It also allows to identify on which batch the trial was conducted, thanks to the root grating date. Batch 1 was grated on 2021/07/06, batch 2 on 2021/06/03 and batch 3 on 2021/06/16. Moreover, the sample name written in the INFO file indicates whether the batch was grated using the grater manufactured by *Gauthier* (median diameter d_50_=1000 µm) or by *Magimix* (d_50_=700 µm). Samples are coded with a majuscule letter representing the grater (i.e. G for *Gauthier*, M for *Magimix*) and the sample number. For example, a sample named “G_42” was grated with *Gauthier* device and is the 42^nd^ packaged bag.

**Fig. 1:**
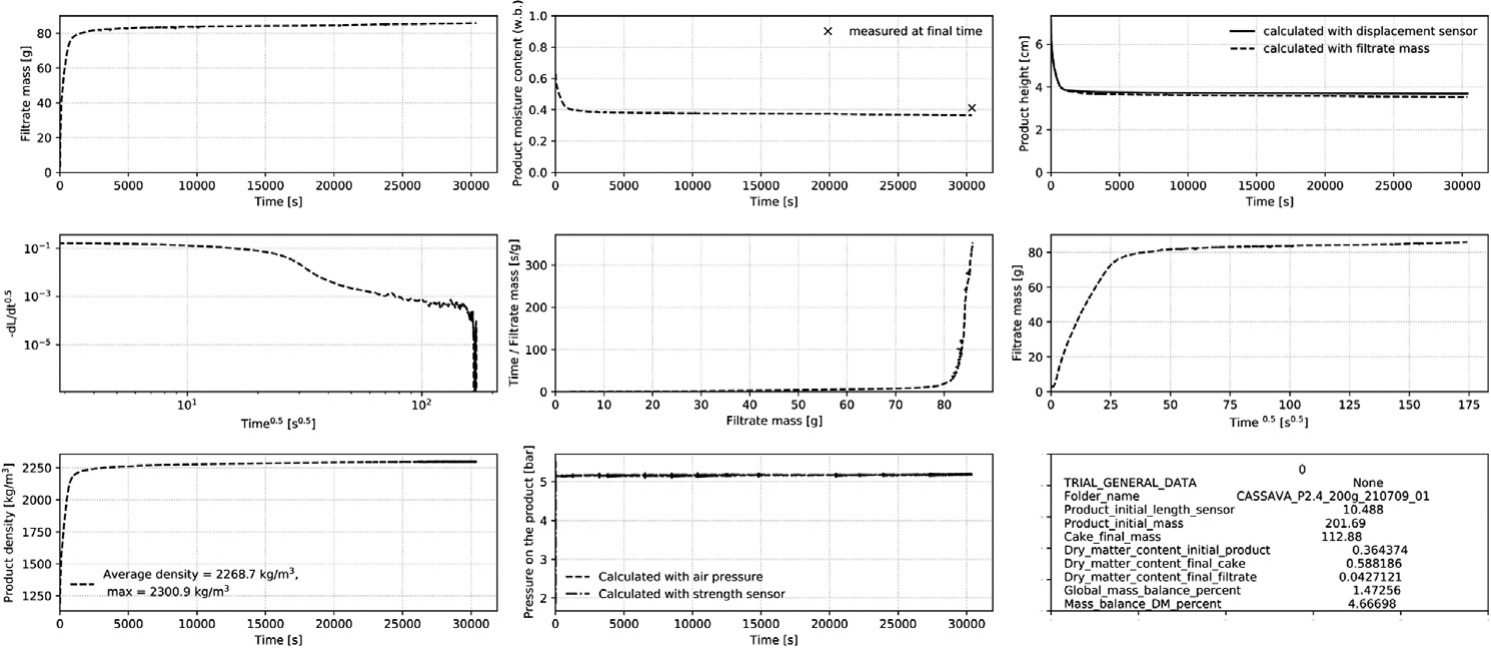
Example of plots generated by the python file to analyze the dewatering kinetics.

A python script, named trial_data_analysis_plots.py, in the repository, reads these files, processes the data, and saves a pdf file with plots and information required to analyze the kinetics (example in Fig. 1). The 3 plots on the top of the figure present the evolution over time of the filtrate mass, the cake moisture content, and the cake height. Redundant measurements are plotted to verify the validity of the kinetics (i.e. final moisture content measured; product height deduced from two experimental measurements). The 3 plots in the middle of the figure are meant to help to identify the transition between filtration and consolidation stages [4]. At bottom of figure, the evolution of product density and of the pressure over time is plotted. The plot of pressure allows to verify the experimental conditions. In addition, main experimental information is written in a box (e.g. product, error on final moisture content). To use the script, the code name of the experimental files has to be conserved.

**Fig. 2:**
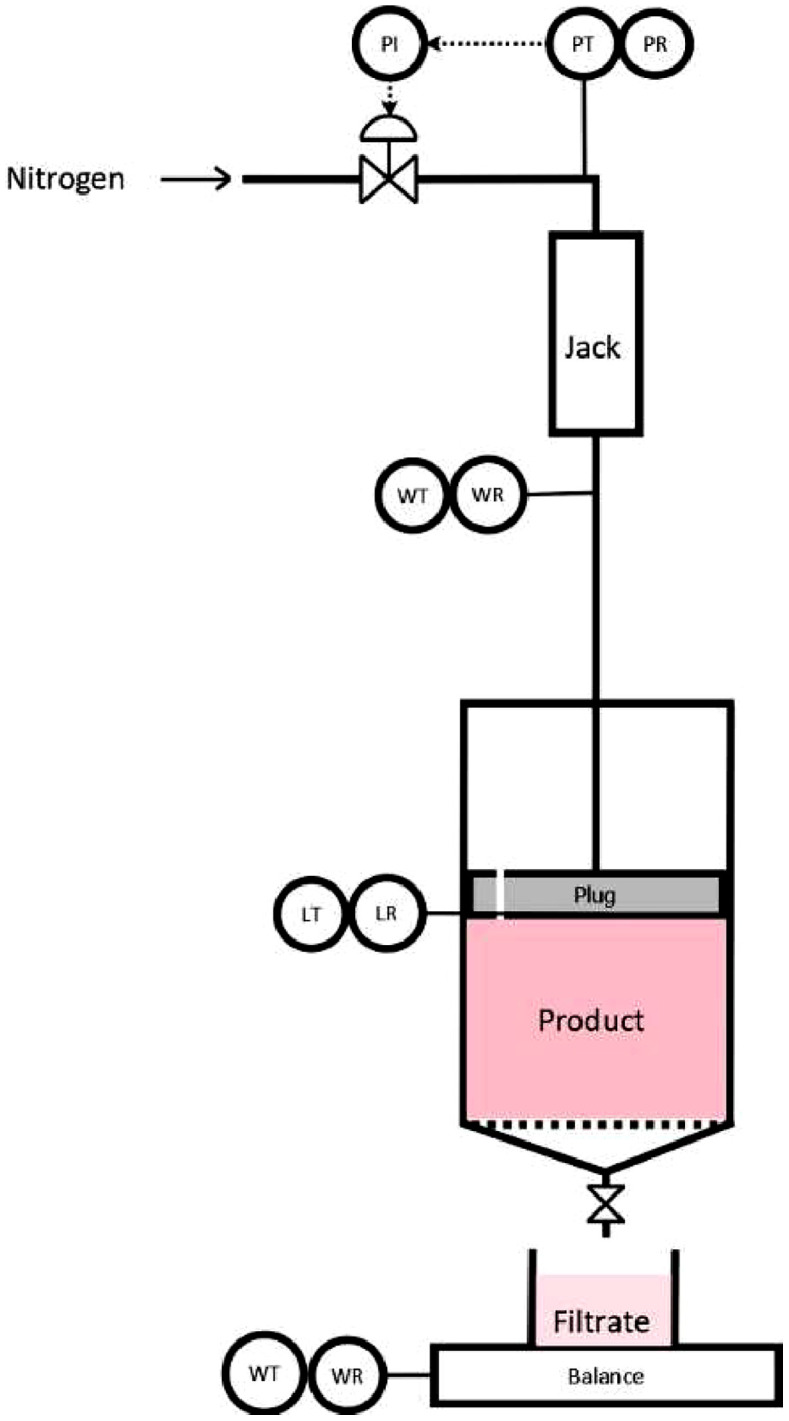
Instrumentation diagram of the compression cell. With LR the length recorder; PI the pressure indicator; PR the pressure recorder; PT the pressure transmitter; WR the strength recorder.

### 3.2 Particle size distributions dataset

Cassava mash boasts a notable level of moisture content (i.e. around 60 % wet basis (w.b.)) as well as a high heterogeneity in particle sizes. PSD measurement by spray sieving was thus recommended [2,3]. Nonetheless, this method is not normalized. 9 protocol variants were tested, varying the water flow rate, the spraying and vibration time and the number of repetitions. Table 6 presents the tested protocols.

For dewatering experiments, cassava roots were grated using two devices, manufactured by *Magimix* and *Gauthier*. Cassava roots quality could have an influence on the grating operation, and thus on the PSD of the mash. To evaluate this influence in the framework of the dewatering study [6], 6 batches of roots were grated independently and their PSD measured. One of these batches was also used for dewatering experiments, i.e. batch 1. The five other batches were prepared specially for PSD measurements and numbered from 4 to 8.

Table 2 presents cassava samples whose PSD was measured, and the protocols applied.

**Table 2:** Summary of rasped cassava samples whose particle size distribution was measured. (Repository: psd_trials_summary.xlsx). Batches are presented in next section.

rasping device	defrost	batch	sample	protocol
Gauthier	yes	1	A	i
Gauthier	no	4	A	vi
Gauthier	no	4	B	vi
Gauthier	no	4	C	v
Magimix	yes	1	A	i
Magimix	no	5	A	ix
Magimix	no	5	B	viii
Magimix	no	5	C	vii
Magimix	no	6	A	iv
Magimix	no	6	B	iii
Magimix	no	6	C	iii
Magimix	no	7	A	ii
Magimix	no	7	B	ii
Magimix	no	8	A	i
Magimix	no	8	B	i

Tables 3 and 4 present the PSDs obtained, respectively for cassava rasped with *Gauthier* and *Magimix* devices. Indeed, they present the fraction of dry matter of the samples listed in Table 3 retained on each sieve, and the fraction drawn into the water. To obtain consistent plots, particles drawn into the water were considered as retained on a sieve of 5 µm mesh size. Excel files used to estimate these PSDs, with the related raw experimental data, are available in the repository (files psd_magimix_experimental_data.xls and psd_gauthier_experimental_data.xls). Experimental data include the wet and dry mass of cassava retained on each sieve, and the dry matter content measurement of the charged water flowing through the sieves.

**Table 3:** PSDs measured on cassava rasped with Gauthier device. (Repository: psd_gauthier_summary.xlsx). With 1A, 4A, 4B, 4C the cassava samples presented in Table 3.

Sieve mesh size (µm)	Dry matter fraction retained on each sieve (%)			
	1A	4A	4B	4C
3000	11.8	16.2	17.2	20.8
1000	7.7	8.1	9.2	9.6
710	8.6	8	7.5	7.6
425	6.8	7.5	7.9	7.7
212	2.5	2.9	3.3	3.5
106	0.4	0.6	1	0.9
50	0.3	1.2	1.7	1.8
5	618	55.4	52.3	48

**Table 4:** PSDs measured on cassava rasped with Magimix device. (Repository: psd_magimix_summary.xls). With 1A, 5A, 5B, 5C, 6A, 6B, 6C, 7A, 7B, 8A, 8B the cassava samples presented in Table 3.

Sieve mesh size (µm)	Dry matter fraction retained on each sieve (%)										
	1A	5A	5B	5C	6A	6B	6C	7A	7B	8A	8B
3000	1.3	2.1	3.2	2.6	2.5	1	8	4.9	3.7	2.7	3
1000	4.8	7.3	5.4	5.7	1.3	0.8	0.5	6.8	5.1	4.4	5.1
710	20.4	5.2	10.6	9.8	4.3	2	2.1	11.8	11.9	14.1	15.8
425	13.4	5.4	8.6	9.8	12.7	10	12.3	7.6	9	9	9.8
212	5.7	2.9	2.5	3.1	6.2	8.7	7.6	2.5	3.4	2.7	2.9
106	0.6	0.5	0.4	0.4	1.7	3	1.7	0.5	0.5	0.2	0.4
50	0.2	0.1	0	0.2	2.8	2.1	1.9	0.1	0.1	0.2	0.1
5	53.6	76.6	69.3	68.4	68.5	72.3	65.9	65.8	66.4	66.8	62.8

### 3.3 First outcomes

A first use of these experiments is presented in a research paper [6]. Main outcomes are:

• Development and validation of a model predicting cassava flour kinetic in filtration–consolidation depending on the pressure applied and the ratio of dry matter per filtration area. It is usable to provide guidelines of conception of filter-press.
• Identification of the limit dryness, a key parameter of dewatering performance.
• Identification of cassava properties in filtration-consolidation, depending on the applied pressure.
• No significant difference of behavior between the various cassava flours was observed. The same observation was conducted during convective–diffusive drying [10,11].
• Further studies based on the present dataset should provide new outcomes, including the effect of cassava flour characteristics and optimization of dewatering techniques.

## 4 Experimental Design, Materials and Methods

### 4.1 Cassava preparation

Experiments were conducted on cassava roots imported from Cameroon (batch 3) and Costa Rica (all the other batches, for dewatering and PSD experiments). Roots were manually peeled and grated at a particle size close to a d_50_ of 700–1000 µm [3,8]. Mash having a d_50_ of 1000 µm was obtained with a semi-industrial grater *Gauthier* (France). Mash having a d_50_ of 700 µm was obtained with a domestic *Magimix* cooking system 4200 (France) kitchen equipment. Cassava having a short shelf-life, either the experiment was carried out quickly, with pulp stored at 4 °C if necessary; or the pulp was deep-frozen, stored at −15 °C and thawed in a water bath.

The distinction among batches stems from variances in their importation date, importation location, and/or preparation date.

Table 1 summarizes the preparation, conservation, and use of the 8 batches.

### 4.2 Dewatering kinetics

Cassava mashes were dewatered in the filtration–consolidation cell depicted in Fig. 1, developed and fabricated at Cirad Laboratories in Montpellier, France. The cell is a stainless-steel cylinder of 50 mm diameter and 230 mm height. At the bottom, a perforated stainless-steel plate holds a cloth filter of about 15–20 µm. The filter cloth was obtained in a cassava starch plant. The apparatus is controlled using a LabView program, allowing to adjust the pressure applied by the pneumatic piston^2^ on the product (4–21 bar). It records air pressure, piston strength, piston height, and the weight of the filtrate every second.

For experiments with deep-frozen mash, the sample was prior thawed in a bain-marie at 35 °C. For trials with fresh mash, the sample was grated a few hours before the dewatering. The sample thus prepared was inserted in the filtration–consolidation cell. The piston was lowered until touching the mash, before starting compression, at the pressure level defined in the Labview program. The height of mash in the cell and the mass of filtrate released were measured and recorded during the whole cycle. When the variation of filtrate mass over time became negligible (i.e. less than 0.1 g⋅min^−1^), the compression was stopped. The dewatered mash, called filter cake, and the filtrate were weighted. Subsequent measurements were taken to determine their respective dry matter contents.

The moisture contents of the initial mash, the final cake and the filtrate were measured using different methods. For the cake and the initial mash, the sample was placed on a dry aluminum cup and dried for at least 1 day at 105 °C, as recommended by the AOAC [1]. For the filtrate, to avoid measurement errors due to Maillard reactions [9], the sample was first dried at 45 °C under ambient pressure for one day, and next at 70 °C under vacuum for another day.

### 4.3 Data processing and representation

As presented in section “Data description”, data of each dewatering trial are stored in three files, i.e. one with general information, one with dry matter measurement and one with kinetic data recorded by the compression cell. A commented python script is provided to read these files, process the data, and plot them (Table 5).

**Table 5:** Preparation and use of the batches of cassava roots.

Batch	Rasping device		Conservation		Used for	
	Gauthier	Magimix	Fresh	Frozen	Dewatering	PSD
1	x	x		x	x	x
2		x	x	x	x	
3		x	x	x	x	
4	x		x			x
5		x	x			x
6		x	x			x
7		x	x			x
8		x	x			x

Based on the experimental data, the python script estimates the evolution through the time of (i) the product moisture content and density, (ii) the pressure applied on the product, and (iii) the square root of the product thickness to highlight a possible transition between filtration and consolidation stages [4].

**Fig. 3:**
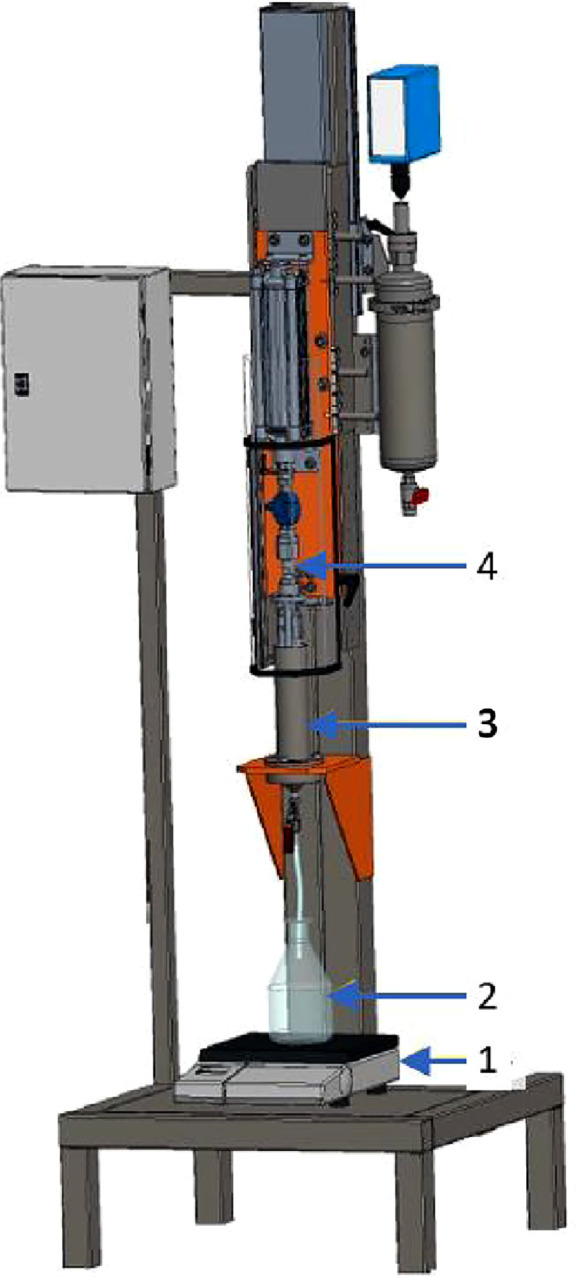
3D image of the compression cell. With (1) the filtrate balance; (2) the filtrate vessel; (3) the cell where the product is filtered and consolidate; (4) the pneumatic piston.

Furthermore, data recorded by the compression cell represent two stages of the experiment: a first stage where the experiment is set up (data not part of the kinetic), and a second stage where the product is dewatered (kinetic data). The second stage begins when the piston reached the product, the pressure setup is reached, and the filtrate valve was opened. The script selects this second stage in the calculation and the plots of the kinetics (Fig. 3).

### 4.4 Particle size distribution

To characterize the particle size distribution, a sieving method was adopted. It consists in passing the product through a series of calibrated sieves stacked on top of each other with decreasing mesh sizes. At the end of the procedure, the weight of product retained on each sieve is measured. Cassava mash was sieved on a stack of seven sieves of decreasing mesh sizes (3000, 1000, 710, 425, 212, 106 and 50 µm) under a water flow for a set time. The pile was then put on a laboratory vibrating sieve machine for another set time. These operations (water spraying and vibration) were repeated a defined number of times (see Table 6). The mass of dry matter retained on each sieve was measured by drying the mash retained at 105 °C for one day. The mass of dry matter smaller than the finest sieve was evaluated by measuring the moisture content of the charged water. Due to the addition of water, the particle size distributions were based on these dry matter .

**Table 6:** Sieving under water spraying protocols applied (available in repository: psd_protocols_summary.xlsx).

Protocol	Protocol step	Flow rate (L/min)	Spraying time (min)	Vibration time (min)	Replicates
ix	1	3.2	5	5	1
ix	2	3.2	2	2	1
ix	3	3.2	3	3	2
viii	1	2.2	5	5	2
viii	2	2.2	2	2	1
viii	3	2.2	3	3	1
vii	1	3.0	5	1	2
vii	2	3.0	4	1	1
vii	3	3.0	5	1	1
vi	1	3.2	5	2	3
v	1	3.2	15	6	1
iv	1	3.0	5	2	1
iv	2	3.2	5	2	2
iv	3	3.2	3	2	1
iii	1	3.2	5	2	3
iii	2	3.2	3	2	1
ii	1	3.0	5	2	3
ii	2	3.0	3	2	1
i	1	3.2	5	2	4

For example in protocol ii: The pulp is positioned on the sieve stack. The sieve stack is put under a water flow of 3L/min for 5 min. The sieve stack is then vibrated for 2 minutes. These last 2 operations are repeated 3 times (sieve under water sprinkling for 5 min then vibration 2 min). The pile is then put under a water flow of 3L/min for 3 min, then on vibration for 2 min. The sieving is then completed. The mass of material retained on each sieve is weighed and the water content of the material measured

To set up the protocol, several conditions of water spraying (flow and time) and vibration (time) were tested (Table 6). To evaluate the repeatability and reproducibility of the rasping procedure and of the PSD measurement, protocol variants were applied on several cassava batches, rasped by two devices (Table 2).

## Limitations

This dataset presents 40 dewatering kinetics measured in a filtration–consolidation cell, on three cassava batches fragmented at two different sizes. It may be completed by kinetics measured on cassava from other origins, eventually fermented, and fragmented at other sizes, to better understand the effect of cassava flour characteristics on its behavior. Kinetics measured in equipment of higher scale or based on other technologies would allow to improve the optimization of the operation.

## Ethics Statement

The authors have read and follow the ethical requirements for publication in Data in Brief. The proposed data does not involve any human subjects, animal experiments, or data collected from social media platforms.

## CRediT authorship contribution statement

**L. Van Der Werf:** Methodology, Conceptualization, Software, Investigation, Writing – original draft, Formal analysis. **A. Chiadò Rana:** Methodology, Software, Investigation, Formal analysis. **A. Chapuis:** Methodology, Conceptualization, Investigation, Funding acquisition. **C. Delpech:** Investigation. **C. Wisniewski:** Methodology, Writing – review & editing. **F. Courtois:** Methodology, Conceptualization, Software, Writing – review & editing, Formal analysis.

## Data Availability

Experimental filtration/compression dewatering kinetics of different cassava flours (Original data) (Dataverse).

## Footnotes

1 The air pressure is not the one applied on the product. Indeed, the air pressure is applied on a jack pushing a piston on the product (see Fig. 2). The calculation of the pressure applied on the product is detailed in the python script.

2 The piston includes a 80 mm diameter jack. This dimension is necessary to estimate the pressure applied on the product with the air pressure.
